# A Biomedical Knowledge Graph System to Propose Mechanistic Hypotheses for Real-World Environmental Health Observations: Cohort Study and Informatics Application

**DOI:** 10.2196/26714

**Published:** 2021-07-20

**Authors:** Karamarie Fecho, Chris Bizon, Frederick Miller, Shepherd Schurman, Charles Schmitt, William Xue, Kenneth Morton, Patrick Wang, Alexander Tropsha

**Affiliations:** 1 Renaissance Computing Institute University of North Carolina at Chapel Hill Chapel Hill, NC United States; 2 Copperline Professional Solutions Pittsboro, NC United States; 3 National Institute of Environmental Health Sciences Durham, NC United States; 4 CoVar Applied Technologies Durham, NC United States; 5 Eshelman School of Pharmacy University of North Carolina at Chapel Hill Chapel Hill, NC United States

**Keywords:** knowledge graph, knowledge representation, data exploration, generalizability, discovery, open science, immune-mediated disease

## Abstract

**Background:**

Knowledge graphs are a common form of knowledge representation in biomedicine and many other fields. We developed an open biomedical knowledge graph–based system termed Reasoning Over Biomedical Objects linked in Knowledge Oriented Pathways (ROBOKOP). ROBOKOP consists of both a front-end user interface and a back-end knowledge graph. The ROBOKOP user interface allows users to posit questions and explore answer subgraphs. Users can also posit questions through direct Cypher query of the underlying knowledge graph, which currently contains roughly 6 million nodes or biomedical entities and 140 million edges or predicates describing the relationship between nodes, drawn from over 30 curated data sources.

**Objective:**

We aimed to apply ROBOKOP to survey data on workplace exposures and immune-mediated diseases from the Environmental Polymorphisms Registry (EPR) within the National Institute of Environmental Health Sciences.

**Methods:**

We analyzed EPR survey data and identified 45 associations between workplace chemical exposures and immune-mediated diseases, as self-reported by study participants (n= 4574), with 20 associations significant at *P*<.05 after false discovery rate correction. We then used ROBOKOP to (1) validate the associations by determining whether plausible connections exist within the ROBOKOP knowledge graph and (2) propose biological mechanisms that might explain them and serve as hypotheses for subsequent testing. We highlight the following three exemplar associations: carbon monoxide-multiple sclerosis, ammonia-asthma, and isopropanol-allergic disease.

**Results:**

ROBOKOP successfully returned answer sets for three queries that were posed in the context of the driving examples. The answer sets included potential intermediary genes, as well as supporting evidence that might explain the observed associations.

**Conclusions:**

We demonstrate real-world application of ROBOKOP to generate mechanistic hypotheses for associations between workplace chemical exposures and immune-mediated diseases. We expect that ROBOKOP will find broad application across many biomedical fields and other scientific disciplines due to its generalizability, speed to discovery and generation of mechanistic hypotheses, and open nature.

## Introduction

“Knowledge graphs” (KGs) have become a common approach for knowledge representation across scientific disciplines, including biomedicine [1]. In a KG, multiple expert-curated “knowledge sources” are integrated into a graph structure, with nodes representing entities and edges providing the relationship between nodes. Within a biomedical KG, the nodes represent biomedical entities, such as *drugs* or *diseases*, and the edges describe relationships that connect the nodes, such as *treats* (eg, *drug treats disease*). The curated knowledge sources that populate a biomedical KG include both databases, such as DrugBank [2] and Comparative Toxicogenomics Database (CTD) [3], and ontologies, such as Monarch Disease Ontology [4] and Human Phenotype Ontology [5]. Various reasoning tools and inferential algorithms are typically applied to KGs [1], thus allowing users to construct complex queries that ask, for example, *if gene X is connected to both chemical exposure Y and disease Z, then the protein product of gene X may represent a potential drug target*. Indeed, successful applications of KGs include drug repurposing [6] and the identification of new drug targets [7].

While KGs, such as Monarch, are openly available, many of the more sophisticated KGs remain proprietary. Perhaps the most well-known proprietary KG is the Freebase-derived Google KG that powers Google’s web search capability [8]. As part of the Biomedical Data Translator program [9-11], we have developed an open KG-based biomedical system termed Reasoning Over Biomedical Objects linked in Knowledge Oriented Pathways (ROBOKOP) [12,13]. ROBOKOP is designed to answer questions such as *what genes are associated with recovery from COVID-19 infection? why is imatinib effective in the treatment of asthma? what biological pathways are associated with stroke-related morbidity?* Note that these questions imply complex mechanistic relationships between terms in the query such as *drug* and *disease*. The ROBOKOP KG is designed to provide answers in the form of putative mechanistic pathways connecting the query terms.

Herein, we provide an overview of ROBOKOP and its real-world application to data derived from the Environmental Polymorphisms Registry (EPR) within the National Institute of Environmental Health Sciences. Specifically, we focused on an EPR study that aimed to explore the impact of workplace exposures on immune-mediated diseases (IMDs) such as asthma, allergy, multiple sclerosis, rheumatoid arthritis, and ulcerative colitis. We first conducted an exploratory analysis of self-reported exposures and IMD symptoms in order to identify significant associations between workplace chemical exposures and IMD. We then used ROBOKOP to (1) validate statistically significant associations by determining whether plausible connections exist within the ROBOKOP KG and (2) propose biological mechanisms that might explain the associations and serve as hypotheses for subsequent testing.

## Methods

### ROBOKOP

**Figure 1:**
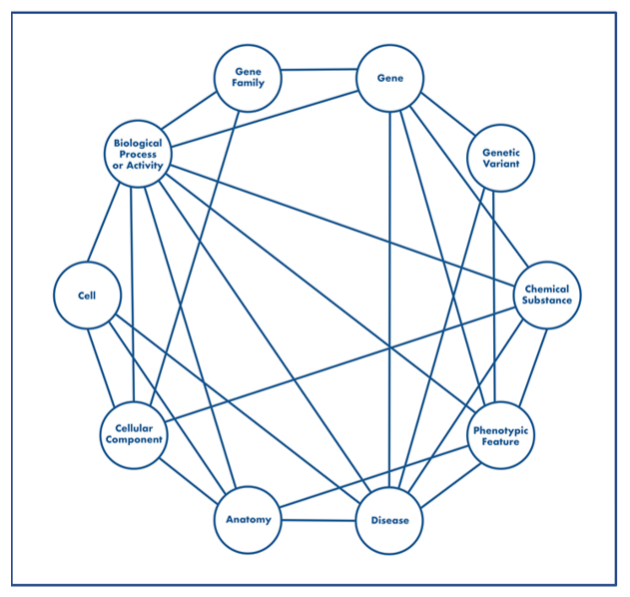
High-level schema for the Reasoning Over Biomedical Objects linked in Knowledge Oriented Pathways (ROBOKOP) knowledge graph, showing classes of nodes as defined by the Biolink model. Note that the schema provides a user guide to assist with the proper construction of queries by providing a visual overview of nodes that are connected in the ROBOKOP knowledge graph.

ROBOKOP is a biomedical KG-based question-answering system that is comprised of both a front-end user interface (UI) and a back-end KG, both of which are openly available [12-15]. The ROBOKOP KG uses the Biolink model [16] as an upper-level ontology that can be applied to express domain knowledge as a graph of relationships between biomedical entities. The ROBOKOP KG currently contains 6 million nodes and 140 million edges, with nodes representing a wide range of biological entities, such as genes, biological processes, anatomical features, diseases, and phenotypes, and edges representing predicates, such as *is associated with*, *causes*, and *increases expression of*. The ROBOKOP KG is derived from over 30 curated biomedical data sources (Multimedia Appendix 1) that have been integrated into a graph structure (Figure 1). The curated data sources were openly available and accessible via direct import into a local Neo4j instance. Some of the data sources were only partially complete and/or required preprocessing.

The ROBOKOP UI allows users to posit queries and quickly explore ranked-and-scored answer sets or subgraphs. ROBOKOP queries are meta-graphs [17-19] or question graphs of the entity types shown in Figure 1. The meta-graph or machine question has a general structure that is defined by the user on the basis of the high-level question of interest. Users can select nodes as named entities specified as a CURIE (Compact Uniform Resource Identifier) or specified only as the entity type. A drop-down menu provides users with choices to free-text node entries. Answers are structured as subgraphs that match the query in topology and type, as well as any desired properties of nodes and edges.

A given query will frequently produce many answers or subgraphs, especially for queries with little specification regarding nodes and edges or with multiple nodes and edges. As such, the ranking of subgraphs by relevance to the query and the strength of the supporting evidence are critical for user exploration of results. The ROBOKOP answer-ranking algorithm [13] weighs each edge within each subgraph using a metric that is based on the number of PubMed abstracts that cite both the source and target nodes. Publication support and predicate assertions are provided by the curated knowledge sources used to create the KG (Multimedia Appendix 1). Additional publication support is provided by a ROBOKOP service termed OmniCorp [20], which contains a graph of PubMed identifiers linked to concepts (ie, potential nodes in the ROBOKOP KG) referenced within abstracts. OmniCorp is built by processing all PubMed abstracts with the SciGraph Named Entity Recognition application programming interface [21] and matching text in titles and abstracts to concepts from a predetermined set of biological ontologies. The ROBOKOP answer-ranking algorithm calculates a confidence score for each answer subgraph with respect to the distance between leaves of the answer subgraph, considering the edge weights as electrical resistance, as defined by Ohm law [22]. Answer subgraphs with greater publication counts will be ranked higher, with publication counts derived from the curated knowledge sources treated with greater importance than those from the publication co-occurrences provided by OmniCorp. The confidence score is then augmented with an “informativeness” score, which is inspired by the NAGA scoring model [23] and treats novel more specific assertions (eg, *disease X interacts with gene* *NPC1*) with greater importance than more generic assertions (eg, *disease X interacts with immune system*).

The ROBOKOP KG can also be queried directly, independent of the UI, using the Cypher query language [24] to find subgraphs within the KG that match the structure of the query. Example Cypher queries can be found online [15].

### Application Use Case Description

The EPR is a study of nearly 20,000 current participants that aims to better understand interactions among environmental exposures and genetic determinants of health and disease [25]. The registry contains survey data on participant exposures and disease history, as well as DNA samples and other biological measurements. As part of the broader effort, investigators have been exploring the impact of workplace exposures on IMDs.

An IMD was defined as a self-reported allergic reaction (allergic rhinitis, hay fever, or seasonal allergies; allergies [other than seasonal]); asthma condition; or autoimmune disorder (psoriasis, thyroid disease [noncancer], hyperthyroidism, hypothyroidism, Crohn disease, multiple sclerosis, celiac disease, Sjogren disease, rheumatoid arthritis, ulcerative colitis, scleroderma or systemic sclerosis, pernicious anemia, myositis, or lupus). EPR survey data were extracted in December 2018. The overall sample size was 4574 participants.

An exploratory analysis was conducted to examine associations between each IMD and specific workplace chemicals classified into one of 18 classes (Multimedia Appendix 2). The survey questions from which the chemicals were obtained were drawn from the EPR “Exposome Survey – Part A: External Exposome, Section B: Chemical and Metal Exposures at Work” and generally structured as follows: “Please select any *heavy metals* you have ever been exposed to *for 15 minutes a week or more* in any job you have held (CHOOSE ALL THAT APPLY).” Associations between workplace chemical exposures and individual IMDs were examined using chi-square analysis or the Fisher exact test, when sample sizes were small due to missing data or few positive cases. A false discovery rate correction was applied to the association tests. Chemicals were examined individually and also by chemical class. Odds ratios (ORs) with lower and upper bounds were calculated per convention and were not adjusted for small sample sizes. As this was an exploratory analysis, the significance level was set at *α*=.05 or .10, and we did not control for potential covariates such as age, sex, and race.

## Results

### Application Use Case Results

A total of 45 exposure-IMD associations were significant at *P*<.10, with 20 associations significant at *P*<.05 after false discovery rate correction. In all cases, workplace chemical exposures were associated with increased odds of self-reported IMD (Multimedia Appendix 3). Dyes were the most common workplace exposure class associated with IMD conditions. No associations were identified between acids or glues/adhesives and IMD conditions. “Allergies or allergic reaction (other than seasonal allergies)” and “allergic rhinitis, hay fever, or seasonal allergies” were the most common IMD conditions associated with workplace chemical exposures.

### ROBOKOP-Derived Mechanistic Assertions Based on Application Use Case Results

We highlight the ROBOKOP application using three exemplar associations that were chosen because they were significant at *P*<.05, evident at both the level of the specific chemical and the chemical class, and representative of different chemical classes and IMDs: (1) carbon monoxide-multiple sclerosis, (2) ammonia-asthma, and (3) isopropanol-allergic rhinitis, hay fever, or seasonal allergies. An overview of ROBOKOP results for each of these examples is provided below, with various functionalities of the interactive UI highlighted in the first example.

#### Carbon Monoxide and Multiple Sclerosis

The association between workplace exposure to carbon monoxide and multiple sclerosis was significant at both the chemical level (OR 6.4583, 95% CI 1.8524-18.2844; *P*=.006) and chemical class level (OR 3.8902, 95% CI 1.2521-10.3546; *P*=.03).

**Figure 2:**
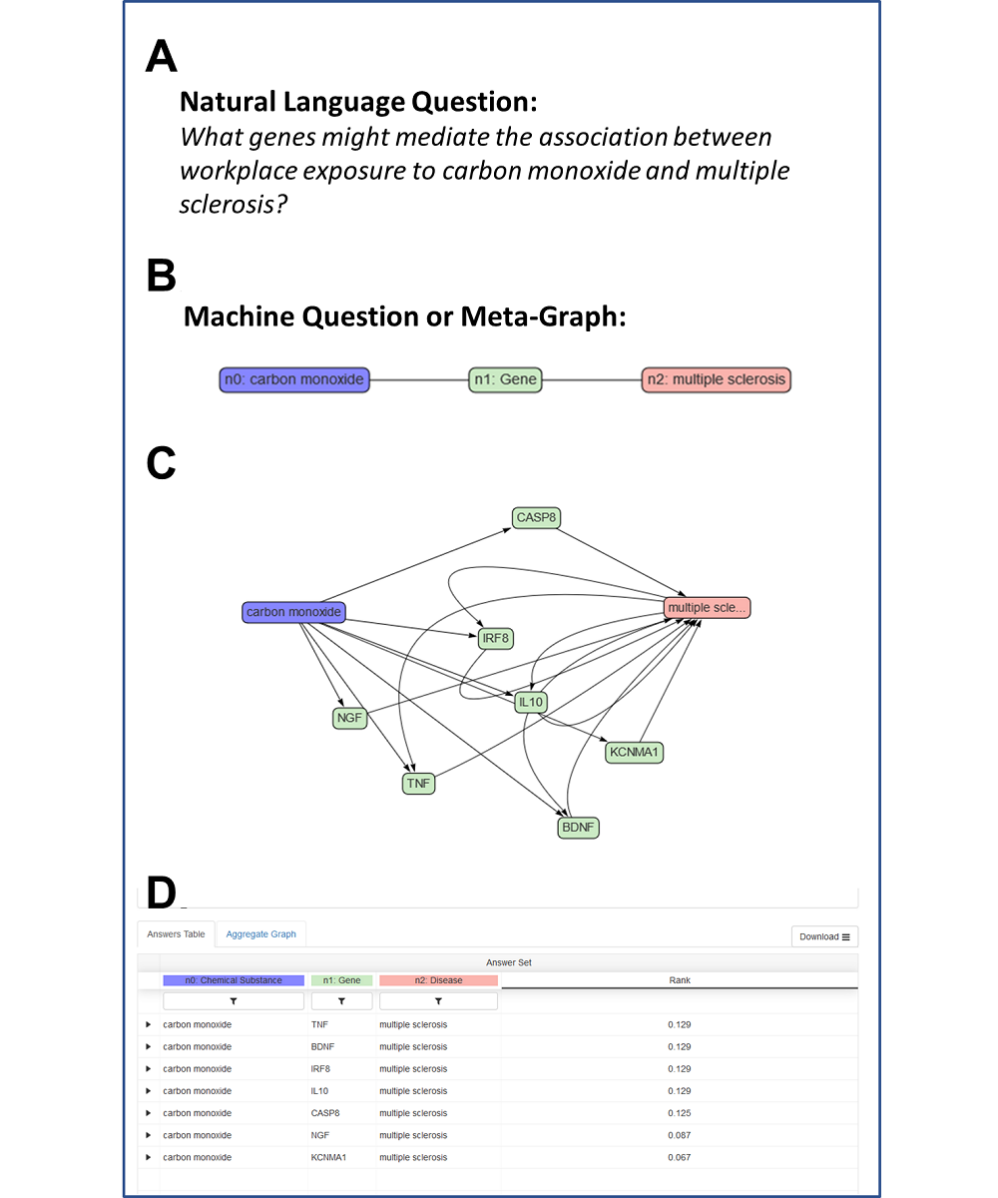
A Reasoning Over Biomedical Objects linked in Knowledge Oriented Pathways (ROBOKOP) high-level question (A) implemented as a machine question or meta-graph (B) designed to explore genes that might mediate the observed association between workplace exposure to carbon monoxide and Environmental Polymorphisms Registry participant self-report of multiple sclerosis. Users select nodes and edges in the ROBOKOP user interface (or by direct Cypher query) to translate the desired natural-language question into an executable machine question, using the schema provided in Figure 1 as a guide. The resultant aggregated answer graph (C) and list of answer subgraphs (D) show six potential mediating genes. Both the answer graph and the list of answer subgraphs are interactive and can be explored by users. For example in (D), users can click on each answer subgraph to explore knowledge sources, predicate assertions, and publication support. TNF: tumor necrosis factor; BDNF: brain-derived neurotrophic factor; IL10: interleukin-10; NGF: nerve growth factor; IRF8: interferon regulatory factor 8; KCNMA1: potassium calcium-activated channel subfamily M alpha 1; CASP8: caspase 8.

We posed the following question to ROBOKOP, but structured as a machine question: *what genes might mediate the association between exposure to carbon monoxide and multiple sclerosis?* The general question, machine question, and answer set are displayed in Figure 2. ROBOKOP identified seven subgraphs and potential intermediary genes as follows: *TNF* (tumor necrosis factor), *BDNF* (brain-derived neurotrophic factor), *IL10* (interleukin-10), *NGF* (nerve growth factor), *IRF8* (interferon regulatory factor 8), *KCNMA1* (potassium calcium-activated channel subfamily M alpha 1), and *CASP8* (caspase 8).

**Figure 3:**
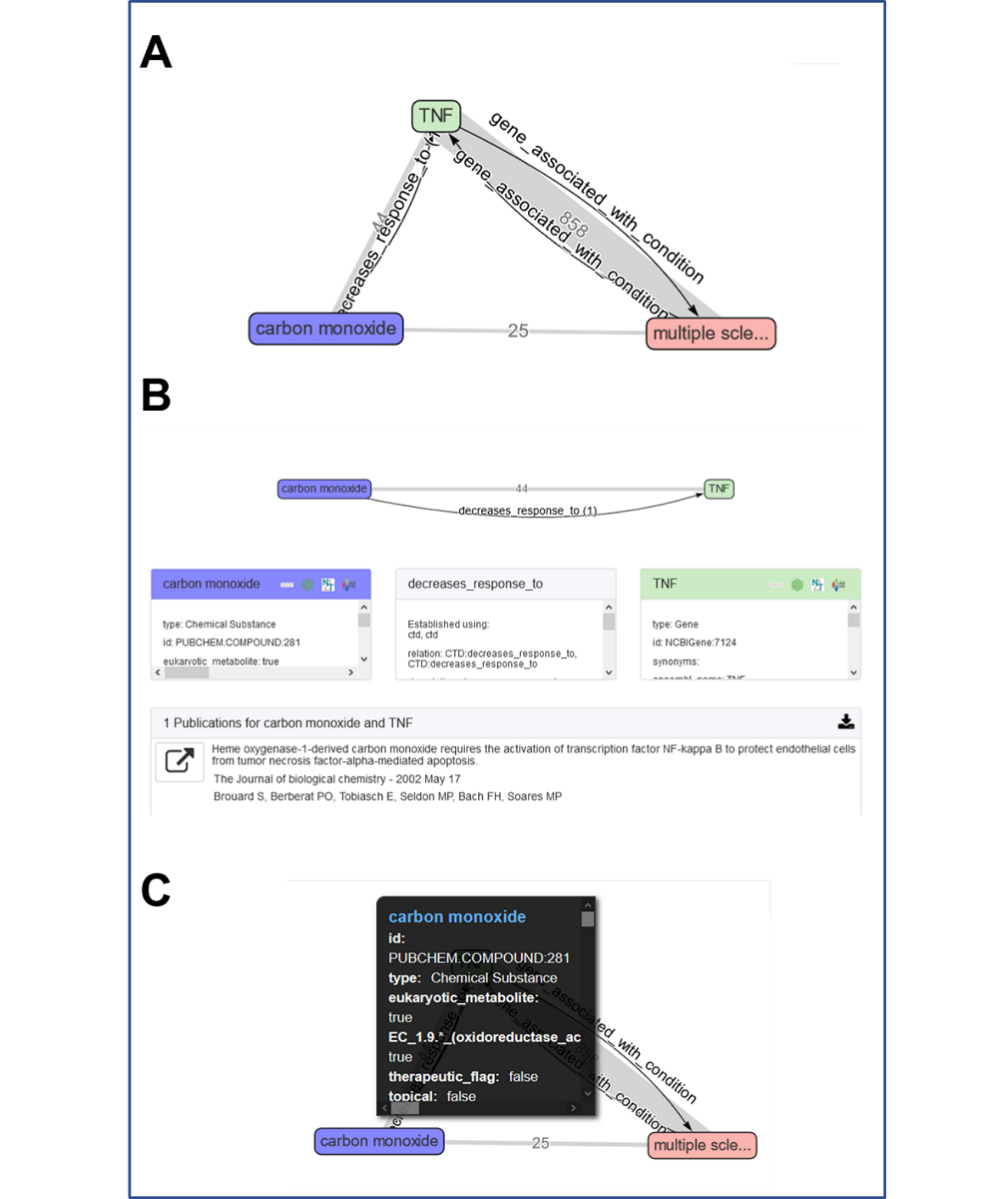
The top-ranked answer subgraph suggesting the involvement of *TNF* (tumor necrosis factor) (A) as a potential gene that might mediate the observed significant association between workplace exposure to carbon monoxide and multiple sclerosis. The Reasoning Over Biomedical Objects linked in Knowledge Oriented Pathways (ROBOKOP) machine question was structured as follows: carbon monoxide – gene – multiple sclerosis. Example publication support for the linkage between carbon monoxide and *TNF* suggesting a role for heme oxygenase-1 (B). Metadata for the carbon monoxide node (C).

The top-ranked answer set had 858 PubMed publications, contributed by OmniCorp, supporting the association between multiple sclerosis and *TNF*, and 44 PubMed publications, again contributed by OmniCorp, supporting the association between carbon monoxide and *TNF* (Figure 3). Twenty-five additional PubMed publications (from OmniCorp) supported an association between carbon monoxide and multiple sclerosis, with several suggesting the involvement of heme oxygenase-1, which is described as an enzyme that oxidizes heme to bilirubin and carbon monoxide. The multiple sclerosis-*TNF* association was established by both HETIO and Pharos. The carbon monoxide-*TNF* association was established by CTD, with publication support contributed by CTD that again suggested a role for heme oxygenase-1 [26].

#### Ammonia and Asthma

The association between workplace exposure to ammonia and asthma was significant at both the chemical level (OR 2.0422, 95% CI 1.4426-2.8524; *P*=.001) and chemical class level (OR 1.5210, 95% CI 1.1938-1.9311; *P=*.004).

The query that was posed to ROBOKOP aimed to identify potential genes that might mediate the association between ammonia and asthma, and it was structured similarly to the query in Figure 2 (ammonia–gene–asthma). ROBOKOP identified nine answer sets or individual paths through the aggregated answer graph. The intermediary genes were *ADA* (adenosine deaminase), *PRKG1* (protein kinase cGMP-dependent 1), *S100B* (calcium binding protein B), *TNF*, *MPO* (myeloperoxidase), *IL6* (interleukin-6), *IL1B* (interleukin-1 beta), *PDE4A* (phosphodiesterase 4A), and *PARP1* (poly(ADP-ribose) polymerase 1).

The lowest-ranked answer subgraph identified *ADA* as the intermediary gene. The metadata associated with *ADA* indicated that ADA metabolizes adenosine by converting it to inosine and ammonia. For this answer subgraph, the relationship between asthma and ammonia was supported by 93 PubMed publications, identified by OmniCorp. An *ADA*-asthma relationship was established using Monarch, with 32 supporting PubMed publications contributed by OmniCorp. Many of the supporting publications suggested a relationship between *ADA* mutations and asthma, allergy, and immune function [27].

#### Isopropanol and Allergic Rhinitis, Hay Fever, or Seasonal Allergies

The association between workplace exposure to isopropanol and allergic rhinitis, hay fever, or seasonal allergies was significant at the chemical level (OR 1.3990, 95% CI 1.1415-1.7155; *P*=.02) and chemical class level (OR 1.2323, 95% CI 1.0287-1.4764; *P*=.09).

As with the other two examples, the query that was posed to ROBOKOP aimed to identify intermediary genes and was structured similarly, except that “allergic disease” was used as the disease node, instead of “allergic rhinitis, hay fever, or seasonal allergies,” because the latter disease node was not available in ROBOKOP.

ROBOKOP identified the following three intermediary genes: *IL6*, *TNF*, and *CSF2* (colony stimulating factor 2). The intermediary gene in the second top-ranked answer set was *IL6*. An isopropanol-*IL6* relationship was established by CTD, with one supporting publication provided by CTD and two additional supporting PubMed publications contributed by OmniCorp. An *IL6*-allergic disease relationship was established by Pharos, with 79 supporting PubMed publications contributed by OmniCorp. Many of those publications [28] suggested the involvement of the innate immune response (eg, basophils, eosinophils, and mast cells), and several publications suggested neuroimmune involvement (eg, “allergic mood” and hippocampal inflammation) [29].

## Discussion

### Summary of Findings and Related Work

We identified 45 significant associations between workplace chemical exposures and IMD conditions in an EPR cohort, with 20 of those associations significant at *P*<.05 and largely unexpected a priori. Statistical evidence for an association, while important, does not establish a causal relationship or provide any insights into underlying mechanisms. Thus, we applied the open ROBOKOP KG system to validate the observed associations by demonstrating plausible connections between exposures and IMD conditions, and we provide mechanistic insights or hypotheses to explain them. We highlighted the following three use case applications: carbon monoxide-multiple sclerosis, ammonia-asthma, and isopropanol-allergic disease.

ROBOKOP identified plausible answer subgraphs to a query structured as carbon monoxide–gene–multiple sclerosis, thus supporting the statistical association between workplace exposure to carbon monoxide and multiple sclerosis. ROBOKOP further identified the gene that encodes TNF, *TNF*, as one of several potential mediating genes. ROBOKOP metadata and publication support suggested that heme oxygenase-1, which oxidizes heme to bilirubin and carbon monoxide, plays an intermediary role [26]. Levels of heme oxygenase-1 are depressed in patients with multiple sclerosis and further depressed during episodes of disease exacerbation [30], and exogenous carbon monoxide or chemicals that release carbon monoxide or induce heme oxygenase-1 appear to be therapeutic in experimental models of multiple sclerosis [31]. These results suggest that heme oxygenase-1, *via* carbon monoxide, has a homeostatic or anti-inflammatory role that might protect against multiple sclerosis or suppress disease exacerbation. However, more recent evidence suggests that a chronic heme oxygenase-1 response in glial cells may promote neurodegeneration and thereby exacerbate multiple sclerosis and other neurodegenerative diseases [32].

The statistical association between workplace exposure to ammonia and asthma was supported by multiple answer subgraphs that were returned by ROBOKOP. The gene that encodes ADA, *ADA*, was identified by ROBOKOP as one of several potential intermediates in this relationship. ROBOKOP metadata indicated that ADA metabolizes adenosine by converting it to inosine and ammonia. ROBOKOP identified publications suggesting an association between *ADA* mutations and asthma, allergy, and immune function, including one publication suggesting an association with aspirin-intolerant asthma [27]. A recent review indicated that ADA deficiency may have detrimental effects on multiple organ systems, including the pulmonary system [33]. Several older publications suggest that adenosine acts as a bronchoconstrictor in persons with asthma [34], and a more recent publication suggested an association between exposure to ammonia and asthma exacerbations [35]. These results support a relationship between *ADA* mutations and pulmonary complications, as well as an association between ammonia and pulmonary complications in persons with established respiratory disease.

ROBOKOP provided several answer subgraphs to support the statistical association between workplace exposure to isopropanol and allergic disease. The gene encoding IL6, *IL6*, was identified by ROBOKOP as one of several potential intermediary genes, with publication support suggesting the involvement of the innate immune response [28]. In addition, ROBOKOP identified publication support suggesting the involvement of IL6 in neuroimmune and neurobehavioral correlates of allergic disease [29]. While IL1 is widely recognized as playing a prominent role in “sickness behavior,” IL6 also appears to play a role [36,37]. These findings suggest that the association between exposure to isopropanol and allergic disease might actually reflect a relationship between isopropanol and neurological/neurobehavioral correlates of allergic disease. Specifically, the results suggest that isopropanol might trigger an innate neuroimmune response that results in elevated levels of IL6, which then might trigger neurobehavioral symptoms of allergic disease.

### Limitations

ROBOKOP has several limitations that should be considered when interpreting the results here or using the application. First, many of the associations between workplace chemical exposures and IMD conditions involved complex chemical mixtures such as “toner,” “transmission fluid,” and “motor oil.” ROBOKOP currently maps these entities to Medical Subject Heading terms, but the application does not have an approach in place for mapping such mixtures to the individual chemicals that constitute a given mixture. We are considering approaches to overcome this limitation. Second, ROBOKOP, like all KG-based applications, is limited by the challenge of KG completion. For example, if an exposure-gene relationship has not been established by one of the curated data sources underlying the ROBOKOP KG, then this relationship will not be identified. We are developing algorithms to overcome this limitation by inferring edges in the KG, but for now, this remains a limitation. Third, evidence for ROBOKOP assertions is derived from the following two main sources: (1) the curated knowledge sources used to create the KG and (2) the co-occurrence of terms in PubMed abstracts. The ranking and scoring algorithm that is used to rank answer subgraphs is based on these two sources of evidence, with the first source treated with greater importance than the second. Other relevant factors, such as date of publication and number of studies on a topic (versus publications), are not considered at present, but may be incorporated into future versions of the application. Fourth, as a prototype system, ROBOKOP does not yet support natural language processing capabilities or other sophisticated approaches to aid users. We encourage users to contact the developers and/or post GitHub issues should they encounter any challenges when generating queries and/or evaluating answer subgraphs. Fifth, while not a limitation of ROBOKOP itself, the workplace chemical exposure–IMD associations reported here were derived from survey data (ie, participant self-report) and were not confirmed by clinical record review or expert judgement. As such, the potential exists for misclassification and/or bias in both the reported exposures and the reported IMDs. Moreover, the timing between workplace exposure and the onset of IMD cannot be determined due to limitations of the survey design and participant recollection at the time the survey was administered. Finally, as this was an exploratory analysis, we did not adjust the ORs for small sample sizes or control for potential covariates such as age, sex, and race.

### Conclusion

ROBOKOP demonstrated potential in its use to support real-world observations and generate mechanistic hypotheses. In this paper, we focused on significant associations between workplace chemical exposures and IMDs, identified as part of a larger EPR study. We note, however, that ROBOKOP has other applications. Indeed, one key feature of ROBOKOP is its generalizability across biomedical domains as a general question-answering system, with capabilities to support a variety of machine questions. For instance, we are using ROBOKOP to explore associations between medications and clinical outcomes, including adverse events, using data derived from electronic health records. We are also testing whether ROBOKOP can be used to support human reasoning on Medical College Admission Tests, and we have promising preliminary results [38]. While the ROBOKOP KG is currently built from biomedical knowledge sources, the general approach is not restricted to the biomedical space. Indeed, on behalf of our institution’s leadership, we are developing a new version of the ROBOKOP KG that is focused on exploring relationships between research proposals and investigator characteristics.

A second key feature of ROBOKOP is its ability to support speed to discovery. For instance, the example use cases presented here took little time to construct and execute, and the interactive UI allows even novice users to posit questions and explore answer subgraphs. The mechanistic insights that were gleaned from the ROBOKOP answer subgraphs were quickly realized, thus allowing for a rapid first-pass analysis of the results and evaluation of the supporting evidence. Moreover, ROBOKOP revealed all potential genes that might mediate the observed exposure-IMD associations via a single query; in effect, the user did not need to spend hours, days, or longer reading through the available literature. This speed to discovery afforded by ROBOKOP also allows investigators to quickly refute associations that may be nothing more than spurious findings (eg, if no answer subgraphs are returned by ROBOKOP). In the examples highlighted herein, we focus on three example exposure-IMD associations. While this may not seem like many, a full literature review to identify potentially novel insights, eliminate spurious findings, and explore supporting evidence would take abundantly more time than was needed to conduct the initial first-pass analysis using ROBOKOP. We plan to leverage ROBOKOP’s speed to discovery in a large-scale analysis of associations between single nucleotide polymorphisms and phenotypes as part of a broader EPR effort and in several other studies.

A third key feature of ROBOKOP is its open nature. Indeed, access to ROBOKOP, whether via the UI or by direct Cypher query of the underlying KG, does not require login authentication or an account; rather, anyone with the URL can access the system. Moreover, the ROBOKOP KG can be downloaded independently of the application [15]. The open nature of ROBOKOP and the multiple routes to access it democratize science and, when coupled with the speed to discovery afforded by the application, should accelerate progress in biomedicine and many other fields.

## Appendix

Multimedia Appendix 1 Reasoning Over Biomedical Objects linked in Knowledge Oriented Pathways (ROBOKOP) knowledge graph data sources.

Multimedia Appendix 2 Workplace chemical exposures explored for their association with immune-mediated diseases.

Multimedia Appendix 3 Significant associations between workplace exposures and immune-mediated diseases, identified as part of the Environmental Polymorphisms Registry.

## Abbreviations

ADA: adenosine deaminase
CTD: Comparative Toxicogenomics Database
EPR: Environmental Polymorphisms Registry
IL6: interleukin-6
IMD: immune-mediated disease
KG: knowledge graph
OR: odds ratio
ROBOKOP: Reasoning Over Biomedical Objects linked in Knowledge Oriented Pathways
TNF: tumor necrosis factor
UI: user interface
