# Supplementary material for: A Biomedical Knowledge Graph System to Propose Mechanistic Hypotheses for Real-World Environmental Health Observations: Cohort Study and Informatics Application
Source: JMIR Med Inform. 2021 Jul 20;9(7):e26714. doi: 10.2196/26714 (PMC8335603; doi:10.2196/26714)
Supplement: Multimedia Appendix 1 [file medinform_v9i7e26714_app1.pdf]

Multimedia Appendix 1. Reasoning Over Biomedical Objects linked in Knowledge Oriented Pathways (ROBOKOP) knowledge graph data sources.

**Data Source**	**Reference**
DrugBank	[1]
DrugCentral	[2]
Aeolus	[3]
Comparative Toxicogenomics Database	[4]
PubChem	[5]
Panther	[6]
UniChem	[7]
ChEMBL	[8]
Chemical Entities of Biological Interest	[9]
mychem.info	[10]
Monarch	[11]
Monarch Disease Ontology	[12]
Human Phenotype Ontology	[13]
Gene Ontology	[14]
QuickGO	[15]
AmiGO	[16]
Pharos	[17]
ClinGen	[18]
ClinVar	[19]
GWAS Catalog	[20]
Kyoto Encyclopedia of Genes and Genomes	[21]
mygene.info	[22]
myvariant.info	[22]
ensembl	[23]
Human Metabolome Database	[24]
UniProt Knowledgebase	[25]
bio2RDF	[26]
MeSH	[27]
ICD-11	[28]
Biolink model	[29]

References

1. Wishart DS, Feunang YD, Guo AC, Lo EJ, Marcu A, Grant JR, Sajed T, Johnson D, Li C, Sayeeda Z, et al. DrugBank 5.0: a major update to the DrugBank database for 2018. Nucleic Acids Res 2018;46(D1):D1074–D1082.
2. Ursu O, Holmes J, Knockel J, Bologa CG, Yang JJ, Mathias SL, Nelson SJ, Oprea TI. DrugCentral: online drug compendium. Nucleic Acids Res 2017;45(D1):D932–D939.
3. Banda JM, Evans L, Vanguri RS, Tatonetti NP, Ryan PB, Shah N H A Curated and standardized adverse drug event resource to accelerate drug safety research. Sci Data 2016;3:160026.
4. Davis AP, Grondin CJ, Johnson RJ, Sciaky D, McMorran R, Wiegers J, Wiegers TC, Mattingly CJ. The Comparative Toxicogenomics Database: update 2019. Nucleic Acids Res. 2019;47(D1):D948–D954.
5. Kim S, Chen J, Cheng T, Gindulyte A, He J, He S, Li Q, Shoemaker BA, Thiessen PA, Yu B, et al. PubChem 2019 update: improved access to chemical data. Nucleic Acids Res. 2019;47(D1):D1102–D1109.
6. Mi H, Muruganujan A, Ebert D, Huang X, Thomas PD. PANTHER version 14: more genomes, a new PANTHER GO-Slim and improvements in enrichment analysis tools. Nucleic Acids Res 2019;47(D1):D419–D426.
7. Chambers J, Davies M, Gaulton A, Hersey A, Velankar S, Petryszak R, Hastings J, Bellis L, McGlinchey S, Overington JP. UniChem: a unified chemical structure cross-referencing and identifier tracking system. J Cheminform 2013;5(1):3.
8. Gaulton A, Hersey A, Nowotka M, Bento AP, Chambers J, Mendez D, Mutowo P, Atkinson F, Bellis LJ, Cibrián-Uhalte E, et al. The ChEMBL database in 2017. Nucleic Acids Res. 2017;45(D1):D945–D954.
9. Hastings J, Owen G, Dekker A, Ennis M, Kale N, Muthukrishnan V, Turner S, Swainston N, Mendes P, Steinbeck C. ChEBI in 2016: improved services and an expanding collection of metabolites. Nucleic Acids Res. 2016;44(D1):D1214–D1219.
10. MyChem.info. https://mychem.info/
11. The Monarch Initiative. https://monarchinitiative.org/
12. Mondo Disease Ontology. http://www.obofoundry.org/ontology/mondo.html
13. Köhler S, Carmody L, Vasilevsky N, Jacobsen JOB, Danis D, Gourdine JP, Gargano M, Harris NL, Matentzoglu N, McMurry JA, et al. Expansion of the Human Phenotype Ontology (HPO) knowledge base and resources. Nucleic Acids Res 2019;47(D1):D1018–D1027.

14. The Gene Ontology Consortium. The Gene Ontology resource: 20 years and still GOing strong. Nucleic Acids Res 2019;47(D1):D330–D338.
15. Binns D, Dimmer E, Huntley R, Barrell D, O'Donovan C, Apweiler R. QuickGO: A web-based Tool for Gene Ontology Searching. Bioinformatics 2009;25 (22):3045–3046.
16. Carbon S, Ireland A, Mungall CJ, Shu S, Marshall B, Lewis S. AmiGO Hub; Web Presence Working Group. AmiGO: Online Access to Ontology and Annotation Data. Bioinformatics 2009;25(2):288–289.
17. Nguyen DT, Mathias S, Bologa C, Brunak S, Fernandez N, Gaulton A, Hersey A, Holmes J, Jensen LJ, Karlsson A, et al. Pharos: collating protein information to shed light on the druggable genome. Nucleic Acids Res 2017;45(D1):D995–D1002.
18. Pawliczek P, Patel RY, Ashmore LR, Jackson AR, Bizon C, Nelson T, Powell B, Freimuth RR, Strande N, Shah N,et al. ClinGen allele registry links information about genetic variants. Hum Mutat 2018;39(11):1690–1701.
19. Landrum MJ, Lee JM, Benson M, Brown GR, Chao C, Chitipiralla S, Gu B, Hart J, Hoffman D, Jang W, et al. ClinVar: improving access to variant interpretations and supporting evidence. Nucleic Acids Res 2018;46(D1):D1062–D1067.
20. Buniello A, MacArthur JAL, Cerezo M, Harris LW, Hayhurst J, Malangone C, McMahon A, Morales J, Mountjoy E, Sollis E, et al. The NHGRI-EBI GWAS catalog of published genome-wide association studies, targeted arrays and summary statistics. Nucleic Acids Res 2019;47 (D1):D1005–D1012.
21. Kanehisa M, Goto S. KEGG: Kyoto Encyclopedia of Genes and Genomes. Nucleic Acids Res. 2000;28(1):27–30.
22. Xin J, Mark A, Afrasiabi C, Tsueng G, Juchler M, Gopal N, Stupp GS, Putman TE, Ainscough BJ, Griffith OL, et al. High-performance web services for querying gene and variant annotation. Genome Biol 2016;17(1):91.
23. Zerbino DR, Achuthan P, Akanni W, Amode MR, Barrell D, Bhai J, Billis K, Cummins C, Gall A, Girón CG, et al. Ensembl 2018. Nucleic Acids Res 2018;46(D1):D754–D761.
24. Wishart DS, Tzur D, Knox C, Eisner R, Guo AC, Young N, Cheng D, Jewell K, Arndt D, Sawhney S, et al. HMDB: the Human Metabolome Database. Nucleic Acids Res 2007;35(Database issue):D521–D526.
25. UniProt Consortium. UniProt: a worldwide hub of protein knowledge. Nucleic Acids Res 2019;47(D1):D506–D515.

26. Callahan A, Cruz-Toledo J, Ansell P, Dumontier M. Bio2RDF release 2: improved coverage, interoperability and provenance of life science linked data. In The Semantic Web: Semantics and Big Data; Springer Berlin Heidelberg, 2013; pp 200–212.
27. Medical Subject Headings. Home Page. 2019. https://www.nlm.nih.gov/mesh/meshhome.html
28. International Classification of Disease. ICD-10. https://icd.who.int/en
29. Biolink model, undated. https://biolink.github.io/biolink-model/
