# Supplementary material for: A Biomedical Knowledge Graph System to Propose Mechanistic Hypotheses for Real-World Environmental Health Observations: Cohort Study and Informatics Application
Source: JMIR Med Inform. 2021 Jul 20;9(7):e26714. doi: 10.2196/26714 (PMC8335603; doi:10.2196/26714)
Supplement: Multimedia Appendix 2 [file medinform_v9i7e26714_app2.pdf]

Multimedia Appendix 2. Workplace chemical exposures explored for their association with immune-mediated diseases.

**Chemical Group**	**Specific Chemicals**
Acids	Hydrochloric acid, sulfuric acid, phosphoric acid, acetic acid, nitric acid, other acid
Alcohols	Isopropanol, methanol, ethanol, butanol, other alcohol
Alkalis	Sodium hydroxide, potassium hydroxide, magnesium hydroxide, other alkali
Anesthetics	Desflurane, enflurane, halothane, isoflurane, methoxyflurane, nitrous oxide, sevoflurane, other anesthetic
Cleaning liquids	Chlorine bleach, ammonia, carbon tetrachloride, other cleaning liquid
Chemical Plastic Production	Bisphenol A, vinyl chloride, styrene, phosgene, phenol, toluene diisocyanate, methylene bis(4 phenyl isocyanate), other compound used in plastic production
Dust	Coal dust, fiberglass dust, rock dust, silica powder, talc, other dust
Dyes	Hair dye, leather dye, textile dye, paper dye, India ink, inkjet printer, gel ink, fountain pen ink, toner, soy ink, pharmaceutical ink, dye ink
Emissions and gasolines	Nitrous oxide, carbon dioxide, monoxide, ozone, other emissions from combustion of gasoline and other fuels
Glues and adhesives	White glue, cement, neoprene, acetate, epoxy, urethane, polyimides, cryanoacrylates, wallpaper paste, adhesive
Heavy metals	Arsenic, beryllium, cadmium, chromates, lead, mercury, nickel, other heavy metal
Lubricants	Brake fluid, fluid, hydraulic fluid, motor oil, oil

Occupational carcinogens	Polybrominated biphenyls, polychlorinated biphenyls, radiation, X-rays, welding fumes, other occupational carcinogens
Pesticides and fumigants	Ethyl dibromide, naphthalenes, insecticides, fungicides, herbicides, ethyl fumigants, rodenticides, fumigants
Paints and paint thinners	Primer, enamel paint and paint thinner, oil-based paint and paint thinner, acrylic paint and paint thinner, luminescent paint and paint thinner, acetone, turpentine, naphtha, methyl ethyl ketone, other paint thinner
Solvents	Benzene, chloroform, chloroprene, dichlorobenzene, ethyl benzene, ethyl dichloride, perchloroethylene, toluene, trichloroethylene, xylenes, solvents and degreasers
Soldering materials	Eutectic (tin-lead alloy), tin-zinc alloy, lead-silver alloy, cadmium-silver alloy, flux, solder paste, solder wire, rosin, other soldering material
Stains and varnishes	Shellac, wood stain, varnish, polyurethane, lacquer, acrylic, other stain or varnish
