# Supplementary material for: A Biomedical Knowledge Graph System to Propose Mechanistic Hypotheses for Real-World Environmental Health Observations: Cohort Study and Informatics Application
Source: JMIR Med Inform. 2021 Jul 20;9(7):e26714. doi: 10.2196/26714 (PMC8335603; doi:10.2196/26714)
Supplement: Multimedia Appendix 3 [file medinform_v9i7e26714_app3.pdf]

Multimedia Appendix 3. Significant associations between workplace exposures and immune-mediated diseases, identified as part of the Environmental Polymorphisms Registry.

Exposure Class and Chemical[†]	IMD Condition	N[‡]	*P* value*	Odds Ratio (Lower Bound, Upper Bound)
**Alcohols**				
Isopropanol	Allergic rhinitis, hay fever or seasonal allergies	3049	**0.022**	1.3990 (1.1415, 1.7155)
**Carcinogens**				
X-rays	Allergic rhinitis, hay fever or seasonal allergies	3049	**0.005**	1.6814 (1.2742, 2.2254)
Radiation	Allergies or allergic reactions (other than seasonal allergies)	3018	**0.034**	1.6225 (1.1885, 2.2092)
**Cleaning Liquids**				
Ammonia	Allergic rhinitis, hay fever or seasonal allergies	3049	**0.003**	1.6885 (1.2910, 2.2143)
Chlorine bleach	Allergies or allergic reactions (other than seasonal allergies)	3018	**0.005**	1.3942 (1.1682, 1.6625)
Ammonia	Asthma	3033	**<0.001**	2.04215 (1.4426, 2.8524)
Chlorine bleach	Asthma	3033	**0.016**	1.5188 (1.1855, 1.9378)
**Dust**				
Fiberglass dust	Rheumatoid arthritis	2622	**0.038**	3.7955 (1.4119, 8.7321)

Talc	Rheumatoid arthritis	2622	0.089	3.3119 (1.2395, 7.5553)
Talc	Myositis	3031	**0.048**	13.5993 (2.3206, 55.9864)
Talc	Allergic rhinitis, hay fever or seasonal allergies	3049	0.057	2.0431 (1.2511, 3.3959)
Talc	Allergies or allergic reaction (other than seasonal allergies)	3018	0.0996	1.9158 (1.1719, 3.1265)
**Dyes**				
Toner	Allergies or allergic reactions (other than seasonal allergies)	3018	**0.018**	1.4131 (1.1491, 1.7354)
Inkjet printer	Allergies or allergic reactions (other than seasonal allergies)	3018	0.022	1.3712 (1.1326, 1.6582)
Fountain pen ink	Allergies or allergic reactions (other than seasonal allergies)	3018	0.097	1.3835 1.0885, 1.7545)
India ink	Allergies or allergic reactions (other than seasonal allergies)	3018	0.068	2.6427 (1.3074, 5.4424)
Toner	Allergic rhinitis, hay fever or seasonal allergies	3049	**<0.001**	1.5776 (1.2908, 1.9300)

Inkjet printer	Allergic rhinitis, hay fever or seasonal allergies	3049	**<0.001**	1.4752 (1.2274, 1.7740)
Fountain pen ink	Allergic rhinitis, hay fever or seasonal allergies	3049	**0.017**	1.4793 (1.1735, 1.8671)
Gel ink	Allergic rhinitis, hay fever or seasonal allergies	3049	**<0.001**	2.0529 (1.4810, 2.8650)
Toner	Psoriasis	3019	0.059	1.8242 (1.1914, 2.7368)
Toner	Asthma	3033	**0.013**	1.6152 (1.2183, 2.1249)
Inkjet printer	Asthma	3033	0.099	1.4305 (1.0942, 1.8579)
**Emissions**				
Carbon monoxide	Multiple sclerosis or MS	3042	**0.006**	6.4583 (1.8525, 18.2844)
Carbon monoxide	Allergies or allergic reaction (other than seasonal)	3018	0.085	1.6943 (1.1524, 2.4828)
Carbon dioxide	Allergies or allergic reaction (other than seasonal)	3018	**0.035**	1.8336 (1.2348, 2.7170)
Carbon monoxide	Allergic rhinitis, hay fever or seasonal allergies	3049	**0.048**	1.7724 (1.2093, 2.6174)
**Heavy Metals**				
Mercury	Celiac disease	3041	0.097	9.1864 (1.6322, 34.9272)

Mercury	Allergies or allergic reaction (other than seasonal allergies)	3018	0.051	1.9780 (1.2379, 3.1579)
**Lubricants**				
Brake fluid	Psoriasis	3019	**0.050**	2.4891 (1.2849, 4.4948)
Motor oil	Psoriasis	3019	**0.028**	2.2455 (1.3127, 3.6819)
Transmission fluid	Psoriasis	3019	0.057	2.5306 (1.2716, 4.6641)
Motor oil	Hypothyroidism (e.g., Hashimoto's thyroiditis)	3026	0.072	0.3927 (0.1836, 0.7476)
**Paints and Paint Thinners**				
Luminescent paint and paint thinner	Allergic rhinitis, hay fever or seasonal allergies	3049	0.051	5.3938 (1.5012, 29.3520)
Naphtha	Myositis	3031	0.093	19.8294 (2.0291, 99.0861)
Oil-based paint and paint thinner	Rheumatoid arthritis	2622	0.066	2.8368 (1.2746, 5.6916)
**Pesticides and fumigants**				
Other pesticide or fumigant	Celiac disease	3041	0.059	27.0539 (2.7595, 134.6697)
**Plastic Production Chemicals**				
Bisphenol A (BPA)	Allergic rhinitis, hay fever or seasonal allergies	3049	0.057	3.3348 (1.4344, 8.6514)
Other compound used in plastic production	Allergies or allergic reactions (other than seasonal allergies)	3018	0.088	4.4087 (1.4075, 16.2313)

Other compound used in plastic production	Asthma	3033	0.086	4.42509 (1.3135, 13.5335)
**Soldering Materials**				
Tin-zinc alloy	Psoriasis	3019	0.097	4.1918 (1.2341,11.3825)
**Solvents and Degreasers**				
Other solvents and degreasers	Allergies or allergic reactions (other than seasonal allergies)	3018	0.058	1.6096 (1.1584, 2.2298)
Dichlorobenzene	Ulcerative colitis	3023	0.072	6.6442 (1.6562,19.5769)
Ethyl benzene	Rheumatoid arthritis	2622	0.057	3.5720 (1.3326, 8.1852)
Ethyl dichloride	Psoriasis	3019	**0.018**	4.2395 (1.5600, 9.9241)

Abbreviations: IMD = immune-mediated disease; OR = odds ratio

†Total N = 4574; Sample sizes for individual associations reflect adjustments for missing data (i.e., no response to survey questions)

‡No significant associations were identified for acids or glues/adhesives

**P values < 0.05 with a false discovery rate correction are in bold font*
